# A framework of artificial light management for optimal plant development for smart greenhouse application

**DOI:** 10.1371/journal.pone.0261281

**Published:** 2021-12-13

**Authors:** João Pereira, Abdul Mounem Mouazen, Mathias Foo, Hafiz Ahmed

**Affiliations:** 1 School of Mechanical, Aerospace and Automotive Engineering, Coventry University, Coventry, United Kingdom; 2 Department of Environment, Ghent University, Ghent, Belgium; 3 School of Engineering, University of Warwick, Coventry, United Kingdom; 4 Nuclear Futures Institute, Bangor University, Bangor, United Kingdom; Ghazi University, PAKISTAN

## Abstract

Smart greenhouse farming has emerged as one of the solutions to global food security, where farming productivity can be managed and improved in an automated manner. While it is known that plant development is highly dependent on the quantity and quality of light exposure, the specific impact of the different light properties is yet to be fully understood. In this study, using the model plant Arabidopsis, we systematically investigate how six different light properties (i.e., photoperiod, light offset, intensity, phase of dawn, duration of twilight and period) would affect plant development i.e., flowering time and hypocotyl (seedling stem) elongation using an established mathematical model of the plant circadian system relating light input to flowering time and hypocotyl elongation outputs for smart greenhouse application. We vary each of the light properties individually and then collectively to understand their effect on plant development. Our analyses show in comparison to the nominal value, the photoperiod of 18 hours, period of 24 hours, no light offset, phase of dawn of 0 hour, duration of twilight of 0.05 hour and a reduced light intensity of 1% are able to improve by at least 30% in days to flower (from 32.52 days to 20.61 days) and hypocotyl length (from 1.90 mm to 1.19mm) with the added benefit of reducing energy consumption by at least 15% (from 4.27 MWh/year to 3.62 MWh/year). These findings could provide beneficial solutions to the smart greenhouse farming industries in terms of achieving enhanced productivity while consuming less energy.

## Introduction

The increase in the global population together with the surge of demand in the food industry has created concerns about food security, prompting a need to explore sustainable agriculture practices to solve this problem [1]. One of the emerging solutions to address this problem is the concept of precision and smart greenhouse farming (see e.g. [2–4]), where the productivity of farming is managed and improved by using technologies involving sensors, actuators and control systems (see e.g. [5–7]). The application of these technologies in smart greenhouse farming has enabled variables such as light, temperature, soil moisture, etc to be controlled and managed to ensure maximum farming productivity can be achieved (see [8] and references therein for more details).

Among all the variables mentioned above, it is known that plant development and growth are greatly influenced by light [9]. Hence, this makes light one of the most important variables that need to be well-managed to ensure good yield and productivity in smart greenhouse farming environment [10, 11]. The evidence of this can be seen with the extensive studies on how to determine the efficient lighting properties for optimal plant growth that is applicable to smart greenhouse farming. In a review paper by Bian et al. [12] the photoperiod, light quality (colours) and intensity have been identified to be the key light properties in regulating plant growth. The authors also discussed the progress and advantages of LED technology to facilitate plant growth in a controlled environment. Nevertheless, due to the different scope of study, the discussion on the quantitative light properties for plant growth is not included. The effect of that same three light properties on the lettuce growth was investigated by Zhang et al., [13]. The authors considered two photoperiods and four different light intensities in their experimental studies and suggested that a 16-hour light/8-hour dark photoperiod with a light intensity of 250 μmol/m^2^s provides the ideal condition for lettuce growth with the lowest energy consumption. In a similar study, Hiroki et al. [14] used the same 16-hour light photoperiod but varied the light period from 18 to 24 hours and showed that a shorter period has the best growth in lettuce albeit at the expense of high energy consumption. On the specific effect of photoperiod on lettuce growth, [15] and [16] both reported that the preferred photoperiod range is between 16- to 20-hour light. The speed breeding method [17] introduced by Watson et al., marks the culmination of the importance of photoperiod in improving crop productivity, where the productivity of crops such as wheat and barleys that are subjected to extended light exposure (up to 22-hour light) have been experimentally shown to accelerate by at least twofold [17]. More importantly, this speed breeding protocol can be readily extended to growth chambers and greenhouses [18], thus making it an attractive avenue for integrating optimal lighting management in smart greenhouse farming for increasing crop productivity.

While significant efforts have been made to identify lighting configuration for maximum plant productivity [19] with lowest energy consumption [20], a systematic framework to provide quantitative optimal lighting recommendations is still lacking. Most studies have been primarily focusing on the effect of light properties like photoperiod, period, and light intensity despite there being other light properties such light offset, phase of dawn, etc. that could also influence plant development. Moreover, as mentioned above, often there is a wide range of these light properties (e.g., photoperiod of 16 to 20 hours) being reported that could improve productivity across different plant species.

The lack of a systematic framework is what forms the motivation of this study. We anticipate that the reported wide range of light properties can be further narrowed down and made more precise through a systematic approach of exploring different aspects of light properties using computational models. The use of computational models can also serve as a complement to existing experimental approaches, which is often time and labour consuming. By providing a more precise quantitative range of these light properties, we could also obtain the added benefit of further energy consumption reduction associated with these lighting operations. This thereby could reduce the operational cost and carbon emission making the food production system more environment friendly (see e.g. [10, 21, 22]).

In this study, using the model plant Arabidopsis, we present a systematic framework of artificial light management across six light properties (i.e., photoperiod, light offset, intensity, phase of dawn, duration of twilight and period) for optimal plant development with the added benefit of improved energy efficiency. This is done by varying these six light properties individually and then collectively using an established mathematical model of the Arabidopsis circadian system relating light input to flowering time and hypocotyl elongation outputs. The circadian system is considered here following the nascent research focus on *circadian agriculture*, where chronobiology is utilised in agriculture for improved food productivity, security and sustainability (see [23] and references therein). The results from this analysis could facilitate better decision making for smart greenhouse farming practitioners with a more specific quantitative range of light properties, which at the same time is able to reduce energy consumption.

The main contributions of this study are as follows: providing for the first time a systematic analysis on the effect of six different light properties, (i.e., photoperiod, light offset, intensity, phase of dawn, duration of twilight and period) on plant development and recommending the best combination of light properties that not only ensures optimum plant development but with minimum energy consumption.

## Materials and methods

### Input light function

Most of the modelling of plant circadian literature uses a simple binary representation, i.e. ‘1’ for ON and ‘0’ for OFF to represent a light function. This binary representation is an adequate representation of the light function used in smart greenhouse farming as light usually turns on and off almost instantaneously. Nevertheless, the binary representation has limited light properties to be analysed. Therefore, in this study, we consider a more comprehensive input light function, which is used in Seaton et al [24] that incorporates six light properties as shown in Eq (1),

$$\begin{array}{cc} L\left(t\right) & =\Delta l+\frac{A}{2}\left\{1+\text{tanh}\left[\left(\frac{P_{r}}{T_{w}}\right)\left(\frac{t+D_{w}}{P_{r}}\right)-\left\lfloor \frac{\left\lfloor t+D_{w}\right\rfloor }{P_{r}}\right\rfloor \right]\right\}\\ & -\frac{A}{2}\left\{1+\text{tanh}\left[\left(\frac{P_{r}}{T_{w}}\right)\left(\frac{t+D_{w}}{P_{r}}-\left\lfloor \frac{\left\lfloor t+D_{w}\right\rfloor }{P_{r}}\right\rfloor \right)-\left(\frac{P_{h}}{T_{w}}\right)\right]\right\}\\ & +\frac{A}{2}\left\{1+\text{tanh}\left[\left(\frac{P_{r}}{T_{w}}\right)\left(\frac{t+D_{w}}{P_{r}}-\left\lfloor \frac{\left\lfloor t+D_{w}\right\rfloor }{P_{r}}\right\rfloor \right)-\left(\frac{P_{r}}{T_{w}}\right)\right]\right\} \end{array}$$ (1)

where *P*_*r*_ is the period, *P*_*h*_ is the photoperiod, *D*_*w*_ is the phase of dawn, *T*_*w*_ is the duration of twilight, *A* is the light intensity (amplitude), Δ*l* is the light offset, tanh is the hyperbolic tangent function and ⌊.⌋ represents the floor function.

### Output phenotype calculation

The two output phenotypes are represented by gene expression of *ATHB2* and *FT* for hypocotyl elongation and flowering time regulatory pathways, respectively. To convert these two gene expressions into measurable outputs, the following equations presented in [24] are used.

The hypocotyl length measured in mm is calculated using

$$\text{Hypocotyl}\;\text{length}=a_{1}\overset{P_{r}}{\underset{0}{\int }}\left(z\left(t\right)-a_{2}\right)dt$$ (2)

where *z*(*t*) is the gene expression of *ATHB2*. If *ATHB2* < *a*_3_, *z*(*t*) = *ATHB2* and if *ATHB2* ≥ *a*_3_, we have *z*(*t*) = *a*_3_. The variable *a*_3_ represents a saturation term to limit the effect of *ATHB2* to further downstream genes within the gene network.

The days to flower measured in days is calculated using

$$\begin{array}{cc} \text{Days}\;\text{to}\;\text{flower} & =d_{0}+\frac{a_{4}}{1-\frac{FT_{area}}{a_{5}}}\\ FT_{area} & =\overset{P_{r}}{\underset{0}{\int }}FT\left(t\right)dt \end{array}$$ (3)

where *d*_0_ is a parameter that fits a sigmoid function to experimental data used in [25]. The parameters *a*_0_ to *a*_5_ are estimated from data obtained across different photoperiods [26, 27]. Following [24], we use *d*_0_ = 16.55; *a*_1_ = 0.9; *a*_2_ = 0.03103; *a*_3_ = 0.8; *a*_4_ = -2308.141; *a*_5_ = 0.02. The expression *FT*_*area*_ is the area under the curve for the gene expression of *FT* over a period. Note that in [25], *FT*_*area*_ is approximated by a quadratic equation, whereas in our study, *FT*_*area*_ is calculated using the above equation.

### Simulation model

The simulation model used in this study is from Seaton et al [24]. The Arabidopsis circadian mathematical model incorporating the flowering time and hypocotyl elongation pathways consist of 48 ODEs, where 30 ODEs are associated with the Arabidopsis circadian clock [28] and 18 ODEs are associated with the two phenotype pathways. For the complete ODEs, see Supplementary Information of [24].

The MATLAB scripts used in our simulation are obtained from S1 File of [24], with minor modifications on the data processing and figure plotting to cater to our analysis. The two main MATLAB scripts from S1 File of [24] that are required for the analysis are *light_conditions*.*m* and *simulate_model*.*m*, which simulate the input light function and the output gene expressions of the phenotypes, respectively.

In the original MATLAB script, only three light properties are varied. Here, we modify the MATLAB script such that we can vary the six light properties to obtain the output gene expression of *ATHB2* and *FT* phenotypes simulated using *simulate_model*.*m*. From the two gene expressions of *ATHB2* and *FT*, the hypocotyl length and days to flower are calculated using Eqs (2) and (3) given above using the file *FT_ATHB2_simscript*.*m*. All the simulation files can be downloaded from https://github.com/mathiasfoo/lightmanagement.

### Effective light duration for flowering calculation

The “Effective Light Duration for Flowering” refers to the duration over the period when the light is turned ON across the total number of days to flower. This can be calculated as follows:

$$\text{Effective}\;\text{Light}\;\text{Duration}\;\text{for}\;\text{Flowering}\;\left(\text{h}\right)=\text{Days}\;\text{to}\;\text{Flower}\;\left(\text{h}\right)\times \left(\frac{P_{h}}{P_{r}}\right)$$ (4)

where *P*_*h*_ is the photoperiod and *P*_*r*_ is the period.

### Energy consumption calculation

LED lights used for smart greenhouse farming are usually powered by the LED driver, which converts AC grid voltage to DC voltage. The luminous flux of the LED is controlled by the current [29] and often the light intensity is assumed to be proportional to the current. Here, we consider the LED lights used for smart greenhouse farming are powered by the 500W Mean Well Model RSP-500-48 LED driver. The LED driver provides a constant voltage of 48 V, a current that varies between 0 to 10.5 A and the AC-DC conversion efficiency is 90.5% [30]. With that, following standard energy consumption derivation (see e.g., [31]), the energy consumption for the LED driver can be calculated as follow:

$$E_{c}=\frac{V_{out}I_{out}A(P_{h}/P_{r})\left(N\times 24\right)}{\eta_{LED}\times 10^{6}}\;\left[\text{MWh}/\text{year}\right]$$ (5)

where *V*_*out*_ is the rated output voltage of the LED driver, *I*_*out*_ is the maximum rated current of the LED driver, *A* is the intensity of the LED, *η*_*LED*_ is the energy conversion efficiency of the LED driver, *P*_*h*_ is the photoperiod, *P*_*r*_ is the period, *N* is the number of days and the multiplication by 24 is to convert days to hours.

## Results and discussion

### Mathematical model relating light to plant development

All living beings are embedded with a biological clock called the circadian clock that can synchronise the organism’s biological functions with the 24-hour day cycles. In plants, the circadian system, which governs the optimal coordination of biological timing has been identified to be responsible for most of the plant development [32–34] such as flowering time, hypocotyl elongation, petal opening, roots growth etc. (see e.g. [35, 36]). The nascent circadian agriculture [23] has led plant biologists to look at crop productivity and sustainability from the circadian system perspective, thereby justifying the use of the plant circadian mathematical model in our analysis.

One of the key influential inputs driving the circadian system that affects plant development is light [37, 38] and to complement experimental studies, many plant circadian mathematical models (see e.g. [24, 28, 39–42]) have been developed to provide better insights into the light-plant development mechanisms. In order for us to carry out a systematic analysis of the light management on plant development, we employ a well-established mathematical model of the plant circadian system developed by Seaton et al., [24], as this model is the only known model to date that comprehensively relates plant circadian system to two phenotypes namely, flowering time and hypocotyl elongation, which will act as a proxy for plant development. The dynamical behaviour of all the regulatory genes involved in flowering time and hypocotyl elongation is modelled using Ordinary Differential Equations (ODE) as discussed in Materials and methods section.

**Fig 1:**
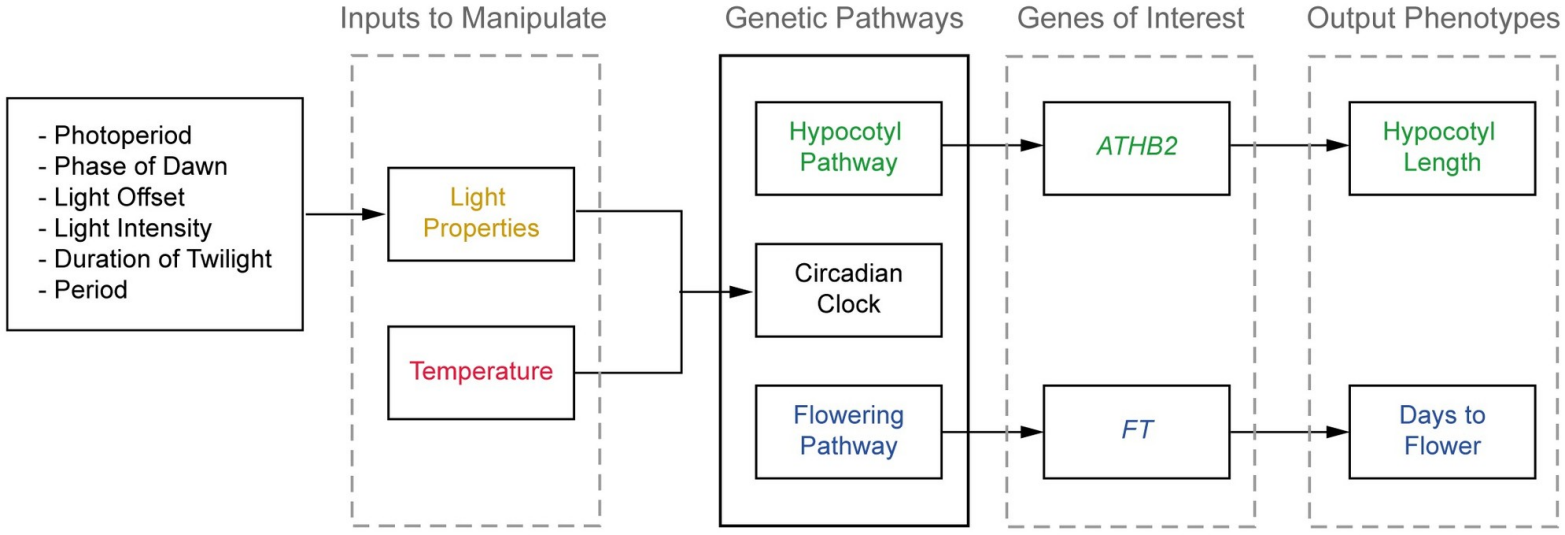
Regulatory pathways relating light input, circadian clock and output phenotypes. The six light properties are varied to study their effect on the output phenotypes viz hypocotyl length and days to flower. The effect of temperature is not investigated in this study, and it is fixed at 22-degree Celsius following [24].

Fig 1 shows the summary of the regulatory genetic pathways relating the input light, circadian system and the two aforementioned phenotypes. The genetic pathways that characterise the plant physiological properties are mathematically modelled taking the input light condition and providing output gene concentrations of *FT* and *ATHB2* that can be converted to days to flower and hypocotyl length respectively (see Materials and methods section). For more details on this model, see [24].

We note that most of the plant circadian mathematical models are developed based on the plant model *Arabidopsis thaliana* grown in a controlled laboratory condition. Although not all knowledge from Arabidopsis can be transferable to crop-based plants grown in the smart greenhouse, the findings from using Arabidopsis can still provide the relevant fundamental knowledge about general plant behaviour [43], thus warranting the validity of using Arabidopsis circadian mathematical model in this study.

The input light function given by Eq (1) used in Seaton et al., is taken from [44], where the light function is governed by six properties, namely photoperiod (i.e., length of light and dark in a period) *P*_*h*_, phase of dawn (i.e., start time of light) *D*_*w*_, light offset Δ*l*, light intensity (amplitude) *A*, duration of twilight (i.e., light/dark transitions regime) *T*_*w*_ and period (i.e., length of one full light cycle) *P*_*r*_. The combination of these six properties results in a distribution of light as a function of time and by individually and collectively manipulating the different properties of the light condition, the role and importance of each property can be well understood.

### Characterisation of nominal phenotypes

To have a reference for comparison in terms of the two phenotypes (days to flower and hypocotyl length), a nominal value for each of the two phenotypes associated with the light function that is commonly used in a typical smart greenhouse environment needs to be first calculated. With that we set the light function (Eq (1)) with the following parameters (Table 1); *P*_*h*_ = 12 h, *P*_*r*_ = 24 h, *T*_*w*_ = 0.05 h, *D*_*w*_ = 0 h, *A* = 1 and Δ*l* = 0. The output gene concentrations of *FT* and *ATHB2* are then converted into days to flower and length in mm using Eqs (2) and (3), respectively following [24]. With these light properties, the nominal value for days to flower is 32.52 days and the hypocotyl length is 1.90 mm (Table 1). By comparing these two nominal values against the one subjected to the variation of different light properties, we can then evaluate which light properties are most influential to plant development. More importantly, we want to determine which of these light properties can be manipulated to improve the two phenotypes viz reduction in days to flower and relatively short hypocotyl length.

**Table 1:** Days to flower and hypocotyl length associated with nominal and recommended light properties.

**Nominal Light Properties**	**Days to Flower (days)**	**Hypocotyl Length (mm)**
Photoperiod, *P*_*h*_ = 12 h Period, *P*_*r*_ = 24 h Duration of Twilight, *T*_*w*_ = 0.05 h Phase of Dawn, *D*_*w*_ = 0 h Light Intensity (Amplitude), *A* = 1 Light Offset, Δ*l* = 0	32.52	1.90
**Recommended Light Properties**	**Days to Flower (days)**	**Hypocotyl Length (mm)**
Photoperiod, *P*_*h*_ = 18 h Period, *P*_*r*_ = 24 h Duration of Twilight, *T*_*w*_ = 0.05 h Phase of Dawn, *D*_*w*_ = 0 h Light Intensity (Amplitude), *A* = 0.99 Light Offset, Δ*l* = 0	21.62	1.18

Flowering time is greatly related to plant development that results in flowers, fruits, and seeds production [45, 46]. Therefore, an increase in flowering time is not preferred as it impedes productivity due to longer generation times for flowers, fruits, and seeds production [47]. The elongation of the hypocotyl is usually advantageous when plants need to compete for more sunlight when growing in a dense environment. Nevertheless, under the absence of competition, a long hypocotyl is disadvantageous as this induces a higher risk of having mechanical damages to the stems of the plants due to the increase of the centre of gravity [48–51]. In the following section, we will vary each light property individually to determine which property can better enable the improvement to the days to flower and hypocotyl length.

### Effect of varying photoperiod

**Fig 2:**
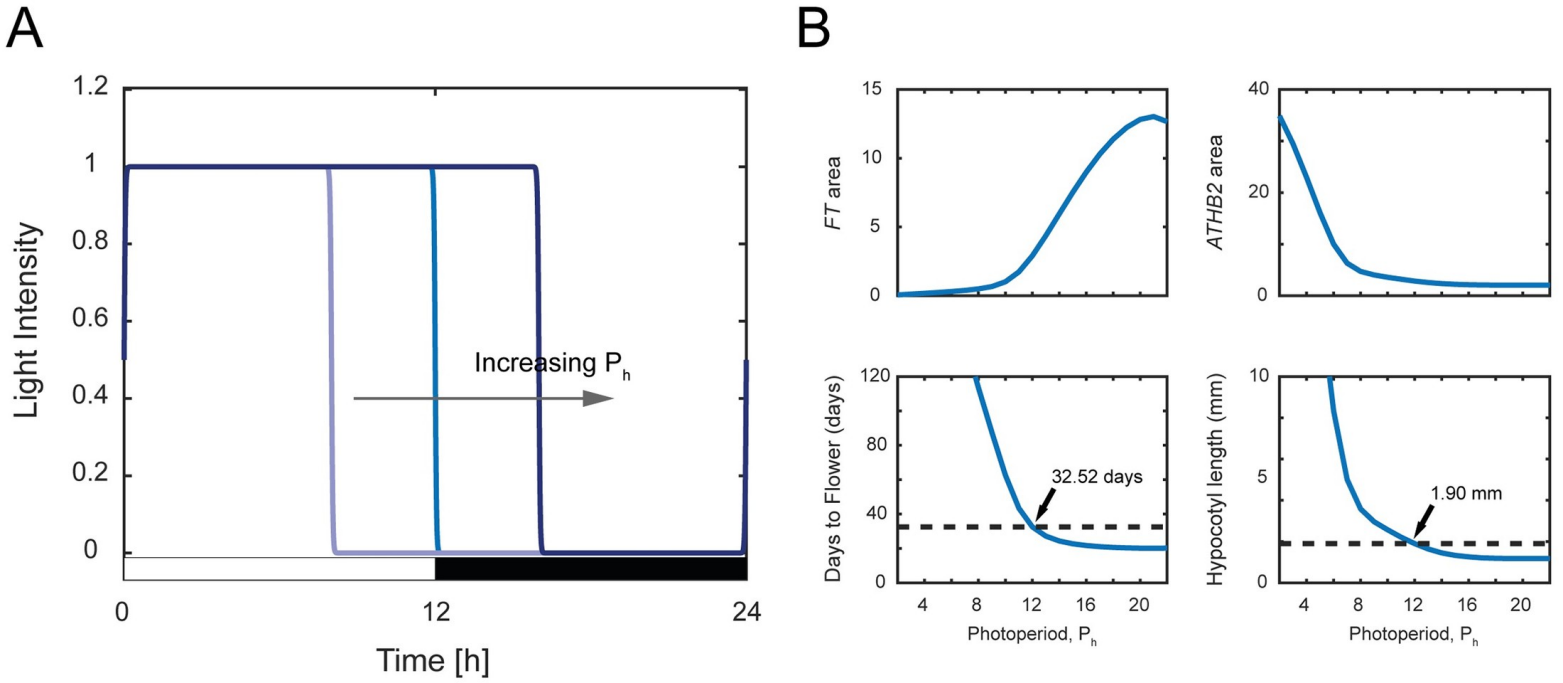
Effect of varying photoperiod *P*_*h*_. (A) Variation of *P*_*h*_ on the light function given by Eq (1). (B) Top row: Simulated gene expressions of *FT* and *ATHB2*. Bottom row: Days to flower and hypocotyl length associated with the simulated gene expressions of *FT* and *ATHB2* calculated using Eqs (2) and (3). The dotted lines represent the nominal value of the days to flower and hypocotyl length.

A photoperiod *P*_*h*_ = 12 h means the input light has 12 hours of light and 12 hours of dark (see Fig 2A). The variation of photoperiod modifies the total hours of light and dark within a 24-hour period. A *P*_*h*_ < 12 h usually represents a short day (e.g., winter season) while a *P*_*h*_ > 12 h usually represents a long day (e.g., summer season). The value of *P*_*h*_ is varied between 0 and 24 hours with increments of 1 hour and the results are shown in Fig 2B and S1 Table of S1 Appendix.

The top row of Fig 2B shows the simulated gene expression levels for *FT* and *ATHB2* while their corresponding hypocotyl length and days to flower calculated using Eqs (2) and (3) are given at the bottom row of Fig 2B. Note that these results are the identical results obtained and shown in [24].

Here, we can clearly see the dependencies of these two phenotypes with their corresponding gene concentrations, where the days to flower are inversely proportional to *FT* expression levels and the hypocotyl length is proportional to *ATHB2* expression levels. For *P*_*h*_ = 0 h and 1 h, we obtain negative values for the days to flower. These negative values occur because the relative expression levels of *FT* are so abnormally low that the second term of Eq (3) becomes more negative than the first term, (i.e., *FT*_*area*_ < *a*_*5*_). Note that *a*_4_ and *a*_5_ in Eq (3) are empirical parameters estimated under the experimental conditions, where the photoperiod ranges from 8 to 24 hours [26]. As the considered photoperiod does not account for a low *P*_*h*_ value, this leads to the negative values of days to flower. Nevertheless, for the purpose of this study, this result is insignificant since the small *P*_*h*_ values correspond to longer days to flower (>100 days), which is undesirable for plant development.

As *P*_*h*_ values increase, we observe the hypocotyl length and days to flower decrease exponentially. The days to flower significantly reduced from 113 days (*P*_*h*_ = 8 h) to 32 days (*P*_*h*_ = 12 h), which corresponds to a drop of 72% with a 50% increase in the number of photoperiods, while the hypocotyl length also significantly drops by almost two-fold from 3.58 mm (*P*_*h*_ = 8 h) to 1.90 mm (*P*_*h*_ = 12 h). By exposing the plant to a longer light (*P*_*h*_ > 16 h) the days to flower and hypocotyl length can be further reduced by 10 days and 0.7 mm respectively. Our analysis shows that *P*_*h*_ = 21 h yields the shortest days to flower with 20.1 days and the hypocotyl length of 1.18 mm. While this *P*_*h*_ = 21 h is close to the one suggested in the speed breeding approach [17], our analysis also reveals that *P*_*h*_ = 18 h would yield very similar phenotype behaviours (i.e. days to flower = 20.6 days and hypocotyl length = 1.19 mm), whilst using up to three hours lesser light per 24 hours period. Utilising three hours less light but with a very similar number of days to flower is favourable as this leads to a reduction of energy consumption (S1 Table of S1 Appendix).

### Effect of varying light offset

**Fig 3:**
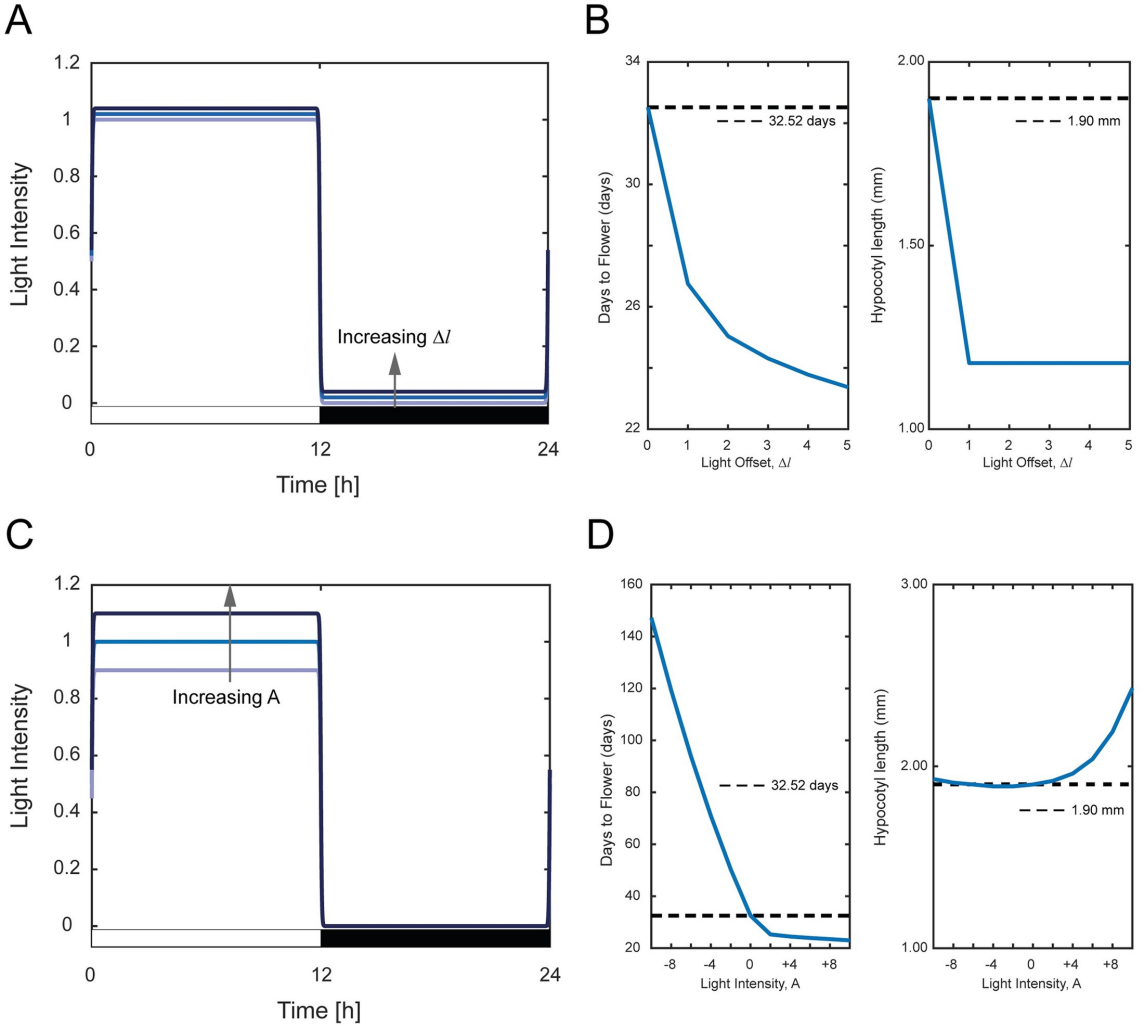
Effect of varying light offset Δ*l* and light intensity *A*. (A, C) Variation of Δ*l* and *A* on the light function given by Eq (1). (B, D) Days to flower and hypocotyl length associated with the variation of Δ*l* and *A*. The dotted lines represent the nominal value of the days to flower and hypocotyl length.

The light offset Δ*l* shifts the light function up or down. For Δ*l* > 0, this represents the presence of background light with minimum light intensity (Fig 3A). We vary Δ*l* from 0 to 5% with increments of 1%. Fig 3B and S2 Table of S1 Appendix show the days to flower and hypocotyl length against the change in Δ*l*, with the black dashed line in Fig 3B representing the nominal values. We observe that light offset brings improvement to both phenotypes. The days to flower decreases exponentially and a change in Δ*l* from 0 to 1% can reduce the days to flower by ~6 days (i.e., from the nominal value of 32.5 days to 26.7 days). Further increase in Δ*l* does further reduce the days to flower albeit not by much. Our variation of Δ*l* ends at 5% because for Δ*l* > 5%, the days to flower remain close to 23 days. For hypocotyl length, only at the onset of Δ*l* do we see changes in the hypocotyl length from the nominal value of 1.90 mm to 1.18 mm at Δ*l* = 1%. Further increase in Δ*l* does not affect the hypocotyl length due to plants being consistently exposed to minimal background light thereby negating the need for the plant to elongate its hypocotyl to seek the light. While Δ*l* = 0.05 yields the best phenotypic values, we recommend Δ*l* = 0.04 instead as the difference in the phenotypes is minimal, at the same time, could potentially reduce energy consumption due to a lower background light intensity.

### Effect of varying light intensity (amplitude)

The light intensity (amplitude) *A* (Fig 3C) corresponds to the amount of photosynthetic photon flux density (PPFD) being received by the plant [52]. We vary *A* within ±10% from the nominal value and the results are shown in Fig 3D and S3 Table of S1 Appendix. Like before, the dashed line represents the nominal values for the two phenotypes. Similarly, to the effect of varying light offset, light intensity is shown to be influential to the days to flower in an exponential manner. For lower light intensities (*A* < 1), the days to flower are significantly larger (i.e., > 50 days) compared to the case of higher light intensities (*A* > 1), where the days to flower ranges between 23–25 days. For *A* < 1, the days to flower increase approximately 20 days for every 2% decrease in the light intensity from the nominal value. For *A* > 1, the days to flower initially reduced by approximately 8 days from the nominal value but then remain unchanged with the increase of light intensity, which is in agreement with some of the reported findings that there is a limit on how much light intensity can affect the circadian clock and its downstream phenotypes (see [53] and the references therein). On the other hand, the hypocotyl length does not seem to be greatly affected by light intensity as we observe the hypocotyl length ranges between 2.0–2.4 mm with *A* varied within ±10%, which is consistent with the findings in [54]. Based on these analyses, we recommend an increase of light intensity by 6% to provide benefits to plant development albeit requiring higher energy consumption due to the increase in light intensity (see e.g., [55]).

### Effect of varying phase of dawn

**Fig 4:**
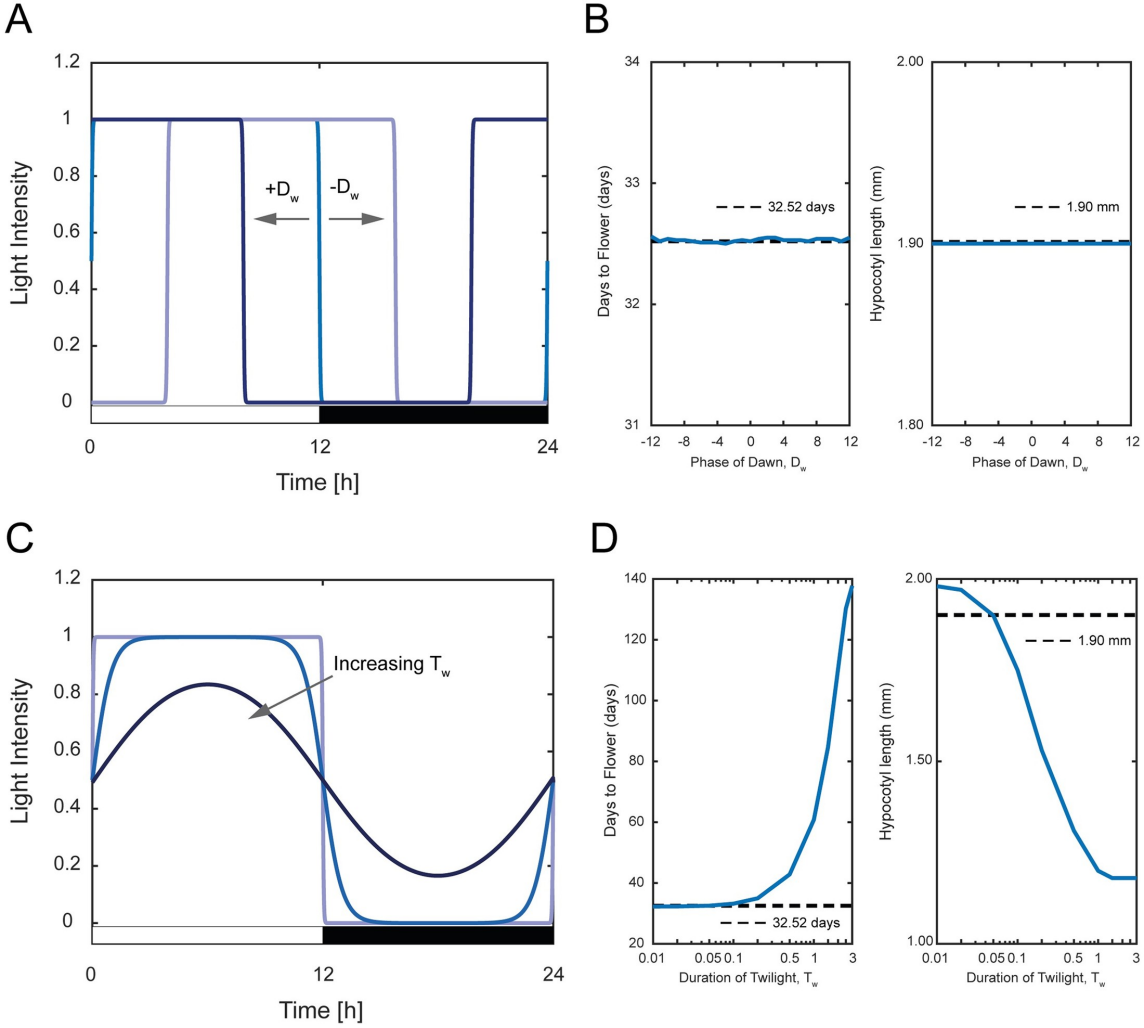
Effect of varying phase of dawn *D*_*w*_ and duration of twilight *T*_*w*_. (A, C) Variation of *D*_*w*_ and *T*_*w*_ on the light function given by Eq (1). (B, D) Days to flower and hypocotyl length associated with the variation of *D*_*w*_ and *T*_*w*_. The dotted lines represent the nominal value of the days to flower and hypocotyl length.

The phase of dawn, *D*_*w*_ is the time when the light first shines. As shown in Fig 4A, varying *D*_*w*_ affects the start position of the light function with *D*_*w*_ > 0 h and *D*_*w*_ < 0 h, respectively, representing a later and earlier start position of the light function. Notably, varying *D*_*w*_ does not alter the shape of the light function but only the start position and thus should not have any influence on the two phenotypes. In Fig 4B, we plot the days to flower and hypocotyl length for different values of *D*_*w*_, with the dashed line representing the nominal values with *D*_*w*_ = 0 h. As expected, there is no significant change in the days to flower and hypocotyl length across different values of *D*_*w*_ (S4 Table of S1 Appendix). The shape of *FT* and *ATHB2* expression levels are practically identical but have their peak expressions at different times of the day (S1 Fig of S1 Appendix). While these expression levels translate into no change in days to flower and hypocotyl length, having different peak expressions could potentially provide other advantages to plants such as temporal coordination of other circadian clock events [42] and reducing cost for protein synthesis associated with waveform generation [56, 57]. Nevertheless, from the perspective of plant development, varying *D*_*w*_ bears no significant impact.

### Effect of varying duration of twilight

The duration of twilight, *T*_*w*_ is related to the decadence of light between extremes of intensity. The increase in the value of *T*_*w*_ results in a slower transition from maximum light to minimum dark (Fig 4C). The variation of *T*_*w*_ also influences the shape of the light function, thereby influencing the total amount of light intensity input to the plant. We vary the value of *T*_*w*_ from 0.01 h to 3 h with a logarithmic increment for *T*_*w*_ < 1 h and linear increment of 0.5 h for *T*_*w*_ > 1 h. Fig 4D shows the two phenotypes plotted against different *T*_*w*_ with the dashed line representing the nominal value. Despite having a significant influence on the days to flower (S5 Table of S1 Appendix), variation of *T*_*w*_ does not reduce the number of days to flower. In fact, the increase in *T*_*w*_ increases the number of days to flower, which is not desirable from the plant development point of view. This is because, with increasing value of *T*_*w*_, reduces the total average amount of light intensity required for the plant to achieve earlier flowering. As for hypocotyl length, we observe a decreasing trend with an increase in *T*_*w*_ prior plateauing to a constant value of 1.18 mm (S5 Table of S1 Appendix). This is because an increase in *T*_*w*_ also introduces background light as this causes the light presence to linger towards the dark cycle that resulting in a behaviour similar to introducing light offset. These results indicate that *T*_*w*_ should not be altered from the perspective of plant development.

### Effect of varying period

**Fig 5:**
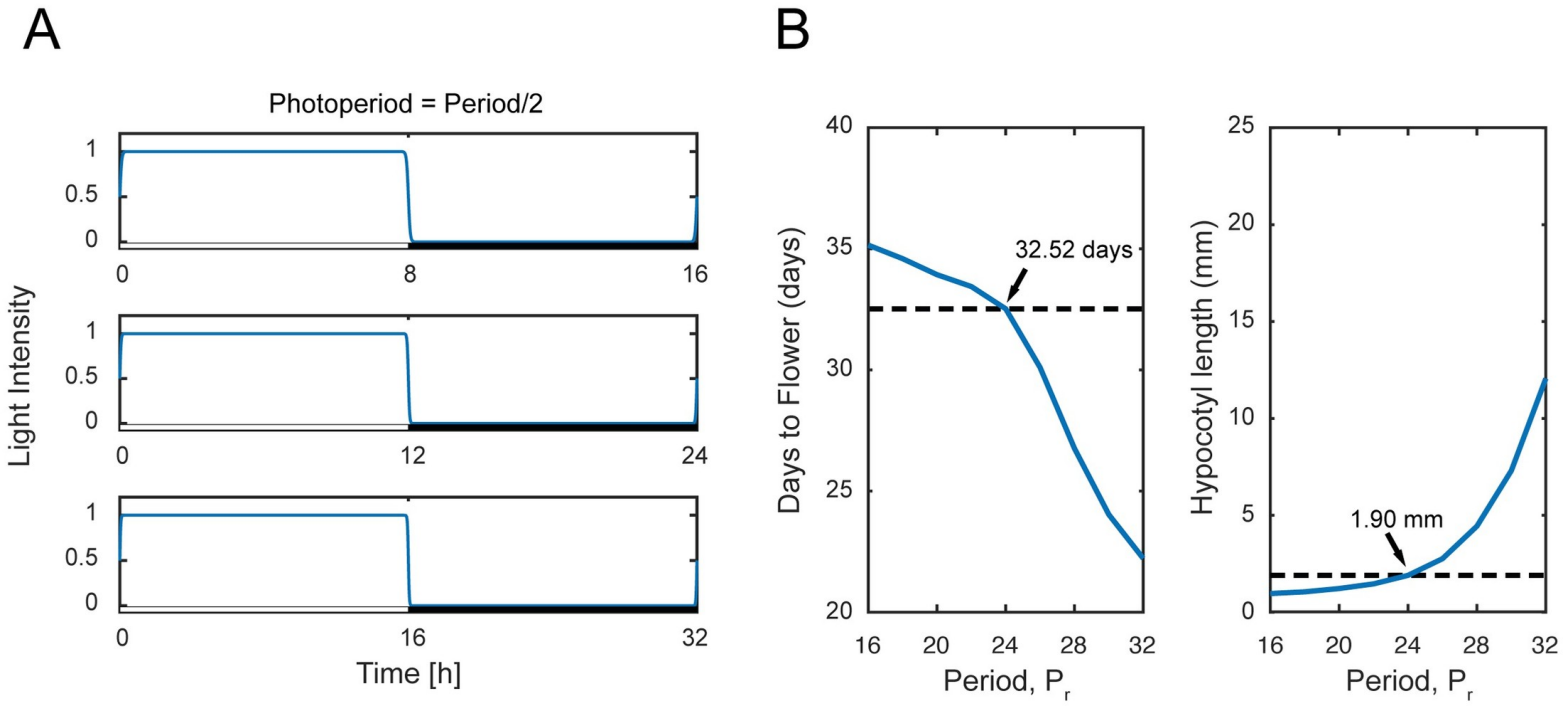
Effect of varying period *P*_*r*_. (A) Variation of *P*_*r*_ on the light function given by Eq (1). (B) Days to flower and hypocotyl length associated with the variation of *P*_*r*_. The dotted lines represent the nominal value of the days to flower and hypocotyl length.

The period *P*_*r*_ manipulation corresponds to the modification of the diurnal period, whereby the light cycle will repeat after a particular number of hours instead of the usual 24 h. It has been reported in the literature that plants grow best in an environment that matches the natural 24 h period with an equal light-dark cycle (12L12D), when compared to a plant growing in *P*_*r*_ = 20 h and *P*_*r*_ = 28 h with their respective equal light-dark cycle i.e., 10L10D and 14L14D respectively [55]. Here, we are interested in the effect of varying the period on plant development by considering an extended range of *P*_*r*_ < 20 h and *P*_*r*_ > 28 h. We vary *P*_*r*_ from 16 to 32 hours with 2 hours increment and, in each period, we set *P*_*h*_ = *P*_*r*_/2 h, i.e., there is always an equal amount of light-dark cycle within the considered period. The illustration of the light function is shown in Fig 5A.

While the increase in *P*_*r*_ value to 32 hours reduces the days to flower from nominal value by ~10 days (Fig 5B (left) and S6 Table of S1 Appendix), the hypocotyl length increases by ~10 mm (Fig 5B (right), and S6 Table of S1 Appendix), which is not desirable. For smaller values of (*P*_*r*_ < 24 h), when compared to the nominal values, while no significant change in the hypocotyl length is observed, we notice an increase in the days to flower, which is also not desirable. Taken together, this result indicates that the phenotypes are sensitive to changes in *P*_*r*_ and the recommended *P*_*r*_ is 24 hours, which is in agreement with the finding of [58].

### Effect of collective manipulation of light properties

Having analysed the effect of varying individual light properties, we are now in a good position to investigate the effects of these light properties collectively. The goal here is to find the combination of light input properties that could further improve plant development (i.e., reduced number of days to flower and relatively short hypocotyl length compared to nominal values). From the six light properties analysed previously, the phase of dawn is not sensitive to plant development, while the duration of twilight deteriorates the plant development and thus these two light properties will be retained at their respective nominal values. Next, the collective manipulation will be carried out between the remaining four light properties, and they are done in a 2-stage manner:

- **Stage 1**: Finding the best combination of *P*_*h*_ and *P*_*r*_
- **Stage 2**: Applying variations to *A* and Δ*l* with the optimal combination from Stage 1.

The search for the collective manipulation of *P*_*h*_ and *P*_*r*_ is carried out first because the analyses given above show that these two light properties are most influential to the phenotypes. S2 Fig of S1 Appendix shows the days to flower and hypocotyl length for several practical combinations of *P*_*h*_ and *P*_*r*_, where we focus only on the combinations that produce the days to flower and hypocotyl length that are not exceeding the nominal values of 32.52 days and 1.90 mm respectively.

**Fig 6:**
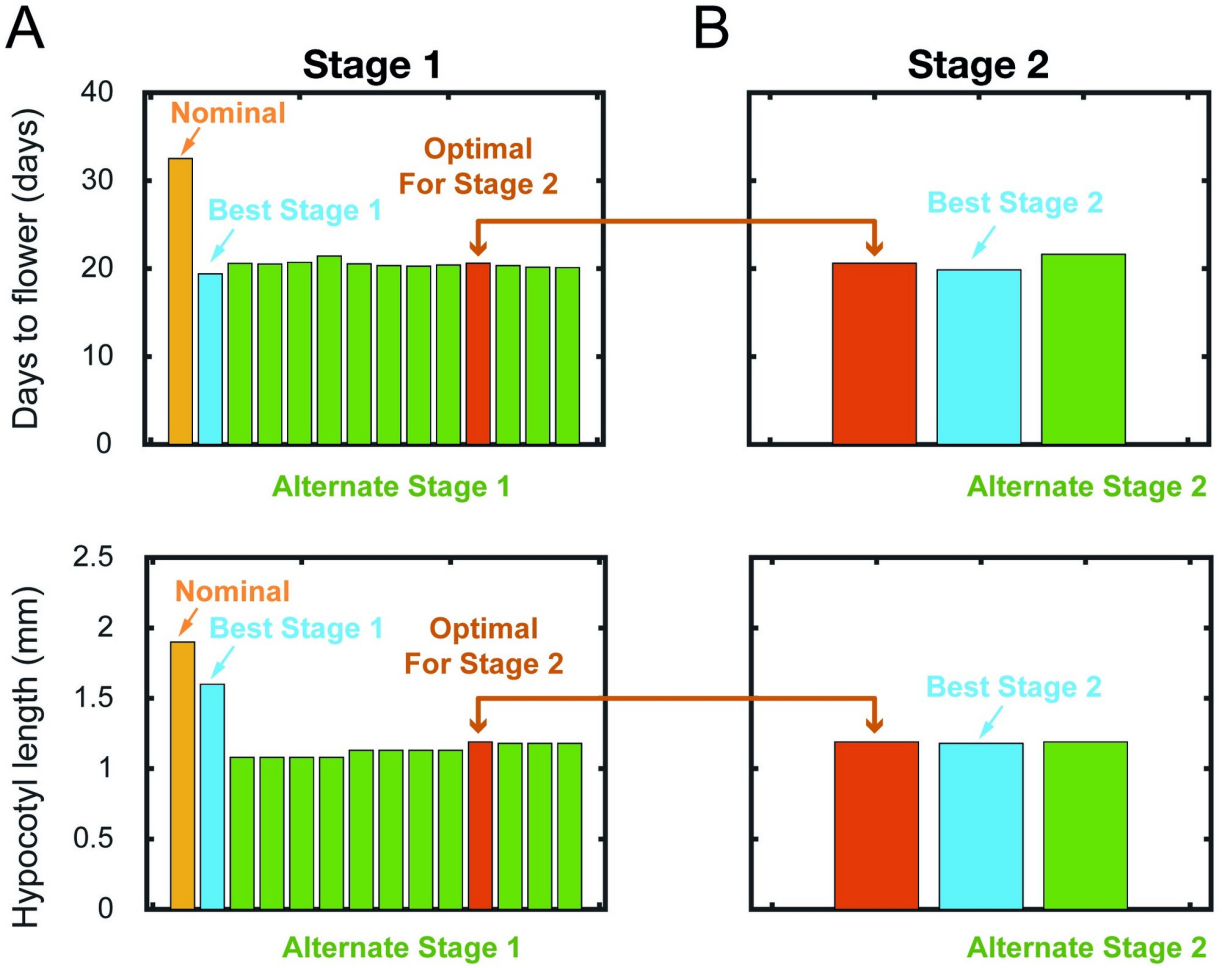
Effect of collective manipulation of light properties. (A) Nominal, best and alternate combinations of *P*_*r*_ and *P*_*h*_ on days to flower and hypocotyl length following S2 Fig of S1 Appendix. (B) Optimal *P*_*r*_ = 24 h and *P*_*h*_ = 18 h from Stage 1, best and alternate combination of *A* and Δ*l* on days to flower and hypocotyl length following S3 Fig of S1 Appendix. The colours used are associated with the same colours used in S2 and S3 Figs of S1 Appendix.

Defining the *P*_*h*_/*P*_*r*_ ratio as the metric to account for the amount of light exposure, where the higher the *P*_*h*_/*P*_*r*_ ratio, the more the plant is exposed to the light per *P*_*r*_ cycle, we see that in general, the trends of the days to flower and hypocotyl length in Stage 1 are as expected, where days to flower and hypocotyl length reduce with the increase in the *P*_*h*_/*P*_*r*_ ratio. The collective manipulation of *P*_*r*_ and *P*_*h*_ enables us to investigate the effect of extended combinations of *P*_*h*_ and *P*_*r*_ on the two phenotypes that are not previously seen when they are individually manipulated. When *P*_*h*_ is manipulated individually, we find that the best phenotypes are obtained with *P*_*h*_ = 18 h with *P*_*r*_ = 24 h. When *P*_*r*_ is manipulated individually, we find that the best value of days to flower is obtained with *P*_*r*_ = 32 h with *P*_*h*_ = 16 h, albeit this induces undesirable hypocotyl length. Interestingly, when considered collectively, due to the wider range and combination of *P*_*r*_ and *P*_*h*_ being considered, we see that *P*_*r*_ = 32 h and *P*_*h*_ = 28 h, produces the best phenotypes in terms of the shortest days to flower of 19.4 days and hypocotyl length of 1.60 mm (Fig 6A and S2 Fig of S1 Appendix), which is respectively, a reduction of ~13 days and 0.3 mm from the nominal values.

Despite *P*_*r*_ = 32 h with *P*_*h*_ = 28 h bringing the best improvement to the two phenotypes, having *P*_*h*_ = 28 h means a longer duration of light is required, which may not be beneficial from an energy savings point of view. A detailed look at S2 Fig of S1 Appendix reveals that significant reduction in days to flower can also be achieved with alternate combinations with *P*_*r*_ between 22 to 24 hours and *P*_*h*_ between 18 to 21 hours, where the days to flower is ~20 days and the hypocotyl length is ~1.13 mm. Comparing these alternate combinations with *P*_*r*_ = 32 h with *P*_*h*_ = 28 h, (Fig 6A and S2 Fig of S1 Appendix) we see an almost negligible difference in terms of the phenotype values but a substantial reduction in the photoperiod, from 28 to 18 hours, which could account for substantial energy savings. We will further discuss this in the next section where we introduce the concept of Effective Light Duration for Flowering. Additionally, having light with *P*_*r*_ > 24 h is not a natural light cycle compared to *P*_*r*_ = 24 h and this may affect other phenotype developments (see e.g. [59]). In view of this coupled with a detailed inspection of S2 Fig of S1 Appendix, the recommended optimal combination from Stage 1 is *P*_*r*_ = 24 h and *P*_*h*_ = 18 h.

Following Stage 1, we set *P*_*r*_ = 24 h and *P*_*h*_ = 18 h and proceed to Stage 2 where we collectively manipulate *A* and Δ*l*. S3 Fig of S1 Appendix shows the days to flower and hypocotyl length for several practical combinations of light intensities and light offsets and we note several interesting observations. In our previous analysis when varying *A* and Δ*l* individually, the improvement in the phenotypes can be achieved with *A* > 1 and Δ*l* > 0. While this trend holds when collective manipulation is considered, introducing light offset seems to be able to improve the phenotypes even when *A* < 1. Achieving improvement with reduced light intensity is desirable as this could help with reducing energy consumption [55]. As shown in Fig 6B and S3 Fig of S1 Appendix, when *A* = 0.99 and Δ*l* = 0.04, we obtain further improvement to the phenotype outputs, with a reduction to the days to flower by ~1 day, while virtually no change in the hypocotyl length.

Having a light offset means the implementation requires a constant background light, which incurs more energy consumption. A detailed look at S3 Fig of S1 Appendix suggests that if we consider *A* = 0.99 and Δ*l* = 0, we increase the days to flower by 1 day but with no change in the hypocotyl length. Whether the energy consumption incurred by having a light offset would be higher than having *A* > 1 depends on various factors that are beyond the scope of this study. Nevertheless, given that the increase in the days to flower is merely by 1 day when *A* = 0.99 and Δ*l* = 0, we are more in favour of these light properties, primarily due to the non-requirement of constant background light due to the light offset.

Based on the findings from both stages of analysis, the recommended light properties (see also Table 1) corresponding to the optimal plant development is given by *P*_*r*_ = 24 h, *P*_*h*_ = 18 h, *A* = 0.99 and Δ*l* = 0 with *D*_*w*_ = 0 h and *T*_*w*_ = 0.05 h. Compared to the nominal value, these light properties improve the days to flower by ~11 days (~34% improvement) and produce shorter hypocotyl length by 0.71 mm (~37% improvement).

### Effective light duration for flowering

Here, we introduce the concept of *Effective Light Duration for Flowering* to aid our choice of light property for optimal output phenotypes with energy savings consideration. As the light function turns ON and OFF across the total number of days to flower, the effective light duration for flowering is defined as duration when only the light is turned ON across the total number of days to flower. The effective light duration for flowering is of particular importance especially when correlating the days to flower with the collective manipulation of photoperiod and period in particular the light exposure, i.e., the *P*_*h*_/*P*_*r*_ ratio.

As an illustration, let us consider the following two configurations of photoperiod and period. In the first configuration, we have days to flower of 32.52 days obtained with *P*_*r*_ = 24 h and *P*_*h*_ = 12 h and for the second configuration, we have days to flower of 24.79 days obtained with *P*_*r*_ = 18h and *P*_*h*_ = 12 h (see also S2 Fig of S1 Appendix). At a first glance, we may think the second configuration is preferred due to the fewer days to flower compared to the first configuration. However, when we compute the Effective Light Duration for Flowering using Eq (4), the duration of light ON are 390.24 hours and 396.64 hours, for the first and second configurations, respectively. Here, we can see that the second configuration in fact uses a longer light duration than the first configuration. This is because despite the two configurations having the same *P*_*h*_ = 12 h, their *P*_*h*_/*P*_*r*_ ratio differs, and this affects the Effective Light Duration for Flowering. By comparing the Effective Light Duration for Flowering for the best and alternate combinations of the *P*_*h*_/*P*_*r*_ ratios given in S2 Fig of S1 Appendix, we, therefore, recommend *P*_*r*_ = 24 h and *P*_*h*_ = 18 h as the preferred light property for optimal plant development with the added benefit of reducing energy consumption (S7 Table of S1 Appendix).

### Recommended light properties and energy consumption

Here, we explore the added benefit from the energy consumption perspective based on the recommended light properties that we suggest in the previous section. The calculation of the energy consumption is given in Eq (5), which incorporate three light properties: photoperiod, period, and intensity. We will compare the energy consumption of the recommended light properties against two other lighting recommendations. The first recommendation follows the speed breeding approach [17] and the second recommendation is based on the combination of light properties that produce the best output phenotypes as given in S2 Fig of S1 Appendix. Table 2 shows the energy consumption for these three recommendations across one year. Our recommendation utilises the least energy consumption with 3.62 MWh/year compared to the speed breeding and best output phenotype where they consume 4.47 MWh/year and 4.27 MWh/year, respectively, which is a respective 19% and 15.1%, reduction in energy consumption. While the calculation of energy consumption given in Eq (5) is not comprehensive, it suffices to demonstrate the added benefit of lower energy consumption based on our recommended light properties.

**Table 2:** Energy consumption for different recommended light properties calculated using Eq (5).

Light Settings	Light Parameters	Energy Consumption
**Recommended Light Properties**	*P*_*h*_ = 18 h *P*_*r*_ = 24 h *A* = 0.99	3.62 MWh/year
**Speed Breeding Approach**	*P*_*h*_ = 22 h *P*_*r*_ = 24 h *A* = 1.00	4.47 MWh/year
**Best Output Phenotype**	*P*_*h*_ = 28 h *P*_*r*_ = 32 h *A* = 1.00	4.27 MWh/year

## Conclusions

The impact of light on plant development has been subjected to extensive studies as evident by the copious literature on this topic. In view of the huge laborious and timely effort, many of those aforementioned studies often could only consider a fraction of light property before making recommendations of the light input. In this study, using a well-established Arabidopsis circadian mathematical model [24], we have systematically investigated the effect of different light properties to develop a framework for artificial light management for optimal plant development for smart greenhouse applications. In particular, we want to determine whether any further improvement on plant development could be achieved by covering a larger range of light properties in our analysis as compared to previous studies and to suggest a quantitative range of light properties with the added benefit of energy savings.

The input light function considered in this study encompasses six different properties, i.e., photoperiod (*P*_*h*_), period (*P*_*r*_), phase of dawn (*D*_*w*_), light intensity (amplitude) (*A*), light offset (Δ*l*) and duration of twilight (*T*_*w*_). We first vary each of the light properties individually while retaining the others and compare the effect with the nominal values of days to flower and hypocotyl length. From the individual light property variation, as expected the most influential light property is the photoperiod as it is associated with the duration of the input light to the plant. On the other hand, the least influential light property is the phase of dawn, where we observe no change in the phenotypes when this property is varied, indicating that the start of light does not affect the phenotypes (see Table 3 for the summary).

**Table 3:** Summary of the influence of each light property on days to flower and hypocotyl length when varied individually.

Light Property	Days to Flower	Hypocotyl Length
**Photoperiod**	Improvements up to 37% (from 32.52 to 20.61 days)	Variation up to 37% (from 1.90 to 1.19 mm)
**Period**	Improvements up to 32% for *P*_*r*_ > 24 h (from 32.52 to 22.2 days)	No improvement but deterioration with *P*_*r*_ > 24 h (from 1.90 to 12.06 mm)
**Light Intensity (Amplitude)**	Improvements up to 24% (from 32.52 to 23.94 days)	Variation up to 7% (from 1.90 to 2.04 mm)
**Light Offset**	Improvements up to 28% (from 32.52 to 23.37 days)	Variation up to 38% at the onset of background light but (from 1.90 to 1.18 mm). No change after the onset of background light.
**Duration of Twilight**	No improvement but deterioration with *T*_*w*_ > 0.05 h (from 32.52 to > 60 days)	Variation up to 38% (from 1.90 to 1.18 mm)
**Phase of Dawn**	No change	No change

The collective manipulation reveals a more complete influence of light properties on the phenotypes. The collective manipulation suggests to us that the light properties that produce the best value of the phenotype are not necessarily the suitable ones for implementation due to potential high energy usage. Our analysis shows that our recommended alternate light properties could also achieve comparable phenotypes values (i.e., days to flower of 21.62 days and hypocotyl length of 1.19 mm) to the best one (i.e., days to flower of 20.61 days and hypocotyl length of 1.19 mm) but with lower energy consumption with savings up to 15%. The outcome of this study would benefit practitioners of smart greenhouse farming by providing them with an efficient way of artificial light management for improved plant development with the added benefit of improved energy consumption.

So far, our analysis involves only white light due to the absence of comprehensive Arabidopsis circadian mathematical model that incorporates different light qualities (colours). There have been many studies reporting the enhancement of plant development when different light qualities are used instead (see e.g. [59–62]). With more smart greenhouse farming begin adopting the use of light qualities in their operation, it would be of great interest to extend our analysis to include the effect of light qualities, which is currently part of our future works.

## Supporting information

S1 Appendix Supporting material: SI tables, SI figures. (DOCX)
