# Supplementary material for: A framework of artificial light management for optimal plant development for smart greenhouse application
Source: PLoS One. 2021 Dec 13;16(12):e0261281. doi: 10.1371/journal.pone.0261281 (PMC8668093; doi:10.1371/journal.pone.0261281)
Supplement: S1 Appendix — (DOCX) [file pone.0261281.s001.docx]

**S1 Appendix: Supporting Material**

**A framework of artificial light management for optimal plant development for smart greenhouse application**

**João Pereira^1^, Abdul Mounem Mouazen^2^, Mathias Foo^3^, and Hafiz Ahmed^4^***

^1^School of Mechanical, Aerospace and Automotive Engineering, Coventry University, Coventry, United Kingdom

^2^Department of Environment, Ghent University, Ghent, Belgium

^3^School of Engineering, University of Warwick, Coventry, United Kingdom.

^4^Nuclear Futures Institute, Bangor University, Bangor, United Kingdom

*Corresponding author

E-mail: hafiz.ahmed@bangor.ac.uk (HA)

**SI Tables**

**S1 Table. Effect of photoperiod on days to flower and hypocotyl length.** *Low *FT* expression that is not captured by the empirical parameters. See main text for details. The effective light duration for flowering is calculated using Equation (4) in the main text.

| Photoperiod, *P_h_* | Days to flower (days) | Days to flower  (hours) | Effective light duration for flowering  (hours) | Hypocotyl length (mm) |
| --- | --- | --- | --- | --- |
| 0 h | Negative  Days* | Negative Hours* | Negative Hours* | 51.82 |
| 1 h | Negative  Days* | Negative Hours* | Negative Hours* | 33.08 |
| 2 h | 1301.26 | 31230.24 | 2602.52 | 30.70 |
| 3 h | 485.58 | 11653.92 | 1456.74 | 25.83 |
| 4 h | 301.47 | 7235.28 | 1205.88 | 19.99 |
| 5 h | 221.86 | 5324.64 | 1109.30 | 13.78 |
| 6 h | 175.21 | 4205.04 | 1051.26 | 8.33 |
| 7 h | 141.35 | 3392.40 | 989.45 | 5.01 |
| 8 h | 113.47 | 2723.28 | 907.76 | 3.58 |
| 9 h | 87.46 | 2099.04 | 787.14 | 2.96 |
| 10 h | 62.59 | 1502.16 | 625.90 | 2.57 |
| 11 h | 43.35 | 1040.40 | 476.85 | 2.21 |
| 12 h | 32.52 | 780.48 | 390.24 | 1.90 |
| 13 h | 27.17 | 652.08 | 353.21 | 1.64 |
| 14 h | 24.34 | 584.16 | 340.76 | 1.45 |
| 15 h | 22.71 | 545.04 | 340.65 | 1.33 |
| 16 h | 21.70 | 520.80 | 347.20 | 1.21 |
| 17 h | 21.04 | 504.96 | 357.68 | 1.19 |
| 18 h | 20.61 | 494.64 | 370.98 | 1.18 |
| 19 h | 20.33 | 487.92 | 386.27 | 1.18 |
| 20 h | 20.16 | 483.84 | 403.20 | 1.18 |
| 21 h | 20.10 | 482.40 | 422.10 | 1.18 |
| 22 h | 20.20 | 484.80 | 444.40 | 1.18 |
| 23 h | 20.57 | 493.68 | 473.11 | 1.18 |
| 24 h | 21.79 | 522.96 | 522.96 | 1.18 |

**S2 Table. Effect of light offset on days to flower and hypocotyl length.**

| Light offset, Δ*l* | Days to flower (days) | Hypocotyl length (mm) |
| --- | --- | --- |
| 0% | 32.52 | 1.90 |
| 1% | 26.75 | 1.18 |
| 2% | 25.04 | 1.18 |
| 3% | 24.31 | 1.18 |
| 4% | 23.78 | 1.18 |
| 5% | 23.37 | 1.18 |

**S3 Table. Effect of light intensity on days to flower and hypocotyl length.**

| Light intensity, *A* | Days to flower (days) | Hypocotyl length (mm) |
| --- | --- | --- |
| -10% | 147.22 | 1.93 |
| -8% | 119.27 | 1.91 |
| -6% | 93.84 | 1.90 |
| -4% | 70.96 | 1.89 |
| -2% | 50.41 | 1.89 |
| 0% | 32.52 | 1.90 |
| +2% | 25.30 | 1.92 |
| +4% | 24.48 | 1.96 |
| +6% | 23.94 | 2.04 |
| +8% | 23.49 | 2.19 |
| +10% | 23.00 | 2.43 |

**S4 Table. Effect of phase of dawn on days to flower and hypocotyl length.**

| Phase of dawn, *D_w_* | Days to flower (days) | Hypocotyl length (mm) |
| --- | --- | --- |
| -12 h | 32.56 | 1.90 |
| -11 h | 32.52 | 1.90 |
| -10 h | 32.54 | 1.90 |
| -9 h | 32.53 | 1.90 |
| -8 h | 32.53 | 1.90 |
| -7 h | 32.52 | 1.90 |
| -6 h | 32.51 | 1.90 |
| -5 h | 32.51 | 1.90 |
| -4 h | 32.51 | 1.90 |
| -3 h | 32.50 | 1.90 |
| -2 h | 32.52 | 1.90 |
| -1 h | 32.53 | 1.90 |
| 0 | 32.52 | 1.90 |
| +1 h | 32.54 | 1.90 |
| +2 h | 32.55 | 1.90 |
| +3 h | 32.55 | 1.90 |
| +4 h | 32.53 | 1.90 |
| +5 h | 32.53 | 1.90 |
| +6 h | 32.53 | 1.90 |
| +7 h | 32.52 | 1.90 |
| +8 h | 32.54 | 1.90 |
| +9 h | 32.54 | 1.90 |
| +10 h | 32.54 | 1.90 |
| +11 h | 32.52 | 1.90 |
| +12 h | 32.55 | 1.90 |

**S5 Table. Effect of duration of twilight on days to flower and hypocotyl length.**

| Duration of twilight, *T_w_* | Days to flower (days) | Hypocotyl length (mm) |
| --- | --- | --- |
| 0.01 h | 32.24 | 1.98 |
| 0.02 h | 32.28 | 1.97 |
| 0.05 h | 32.52 | 1.90 |
| 0.1 h | 33.18 | 1.75 |
| 0.2 h | 34.95 | 1.53 |
| 0.5 h | 42.81 | 1.31 |
| 1.0 h | 60.79 | 1.20 |
| 1.5 h | 84.64 | 1.18 |
| 2.0 h | 110.33 | 1.18 |
| 2.5 h | 130.22 | 1.18 |
| 3.0 h | 137.76 | 1.18 |

**S6 Table. Effect of period on days to flower and hypocotyl length.** For each considered period, the photoperiod is half the period. The effective light duration for flowering is calculated using Equation (4) in the main text.

| Period, *P_r_* | Days to flower (days) | Days to flower  (hours) | Effective light duration for flowering  (hours) | Hypocotyl length (mm) |
| --- | --- | --- | --- | --- |
| 16 h | 35.15 | 843.60 | 421.80 | 0.96 |
| 18 h | 34.59 | 830.16 | 466.97 | 1.05 |
| 20 h | 33.93 | 814.32 | 475.02 | 1.22 |
| 22 h | 33.44 | 802.56 | 434.72 | 1.46 |
| 24 h | 32.52 | 780.48 | 390.24 | 1.90 |
| 26 h | 30.10 | 722.40 | 391.30 | 2.76 |
| 28 h | 26.76 | 642.24 | 374.64 | 4.44 |
| 30 h | 24.03 | 576.72 | 360.45 | 7.31 |
| 32 h | 22.22 | 533.28 | 355.52 | 12.06 |

**S7 Table. Effective light duration for flowering calculated using Equation (4) in the main text for the best and alternate combinations shown in S2 Fig.**

| Best combination | Days to Flower (days) | Days to Flower (hours) | Effective light duration for flowering  (hours) | Hypocotyl length (mm) |
| --- | --- | --- | --- | --- |
| *P_r_* = 32 h, *P_h_* = 28 h | 19.40 | 465.60 | 407.40 | 1.60 |
| Alternate combinations | Days to Flower (days) | Days to Flower (hours) | Effective light duration for flowering  (hours) | Hypocotyl length (mm) |
| *P_r_* = 22 h, *P_h_* = 18 h | 20.59 | 494.16 | 404.31 | 1.08 |
| *P_r_* = 22 h, *P_h_* = 19 h | 20.52 | 492.48 | 425.32 | 1.08 |
| *P_r_* = 22 h, *P_h_* = 20 h | 20.70 | 496.80 | 447.71 | 1.08 |
| *P_r_* = 22 h, *P_h_* = 21 h | 21.43 | 514.32 | 470.09 | 1.08 |
| *P_r_* = 23 h, *P_h_* = 18 h | 20.54 | 492.96 | 386.73 | 1.13 |
| *P_r_* = 23 h, *P_h_* = 19 h | 20.34 | 488.16 | 408.22 | 1.13 |
| *P_r_* = 23 h, *P_h_* = 20 h | 20.27 | 486.48 | 429.70 | 1.13 |
| *P_r_* = 23 h, *P_h_* = 21 h | 20.41 | 489.84 | 451.19 | 1.13 |
| *P_r_* = 24 h, *P_h_* = 18 h | 20.61 | 494.64 | 370.62 | 1.19 |
| *P_r_* = 24 h, *P_h_* = 19 h | 20.33 | 487.92 | 391.21 | 1.18 |
| *P_r_* = 24 h, *P_h_* = 20 h | 20.16 | 483.84 | 411.80 | 1.18 |
| *P_r_* = 24 h, *P_h_* = 21 h | 20.10 | 482.40 | 432.39 | 1.18 |

**SI Figures**


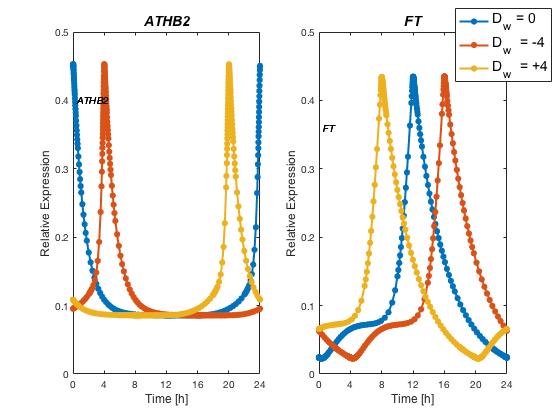


**S1 Fig.** **Simulated gene expressions for *ATHB2* and *FT* under for different values of phase of dawn.** Blue line: Phase of dawn = 0 h (nominal). Red line: Phase of dawn = -4 h. Yellow line: Phase of dawn = +4 h.


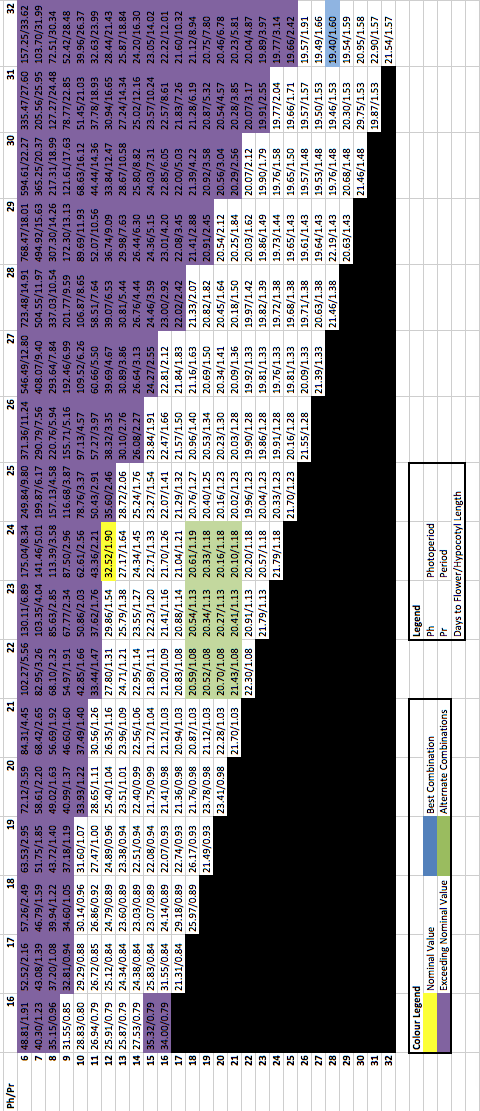


**S2 Fig.** **Effect of collective manipulation of photoperiod and period on days to flower and hypocotyl length (Stage 1).** Each cell contains two values separated by forward slash, where the values of the days to flower and hypocotyl length are shown on the left and right of the forward slash respectively. Purple cell: Values of days to flower and hypocotyl that are worse than the nominal values. Yellow cell: Nominal values. Blue cell: Combination of photoperiod and period that provide the best value of days to flower and hypocotyl length. Green cell: Alternate combination of photoperiod and period that provide value close to the best value of days to flower and hypocotyl length. For more details, see S1 File at <https://github.com/mathiasfoo/lightmanagement>.


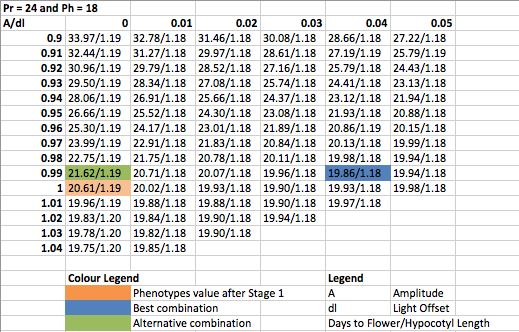


**S3 Fig.** **Effect of collective manipulation of light intensity and light offset with optimal photoperiod and period obtained from Stage 1 on days to flower and hypocotyl length (Stage 2).** Each cell contains two values separated by forward slash, where the values of the days to flower and hypocotyl length are shown on the left and right of the forward slash respectively. Orange cell: Days to flower and hypocotyl length obtained from Stage 1. Blue cell: Combination of amplitude and light offset that provide the best value of days to flower and hypocotyl length. Green cell: Alternate combination of amplitude and light offset that provide value close to the best value of days to flower and hypocotyl length. For more details, see S1 File at <https://github.com/mathiasfoo/lightmanagement>.
